# Demonstrating the Influence of Physical Aging on the Functional Properties of Shape-Memory Polymers

**DOI:** 10.3390/polym10020107

**Published:** 2018-01-23

**Authors:** Ehsan Ghobadi, Mohamed Elsayed, Reinhard Krause-Rehberg, Holger Steeb

**Affiliations:** 1 Institute of Mechanics (CE), University of Stuttgart, 70565 Stuttgart, Germany; Holger.steeb@mechbau.uni-stuttgart.de; 2 Faculty of Natural Sciences II-Chemistry, Physics and Mathematics, Martin-Luther-University Halle, 06120 Halle, Germany; melabdalla@yahoo.co.uk (M.E.); reinhard.krause-rehberg@physik.uni-halle.de (R.K.-R.); 3 Department of Physics, Faculty of Science, Minia University, 61519 Minia, Egypt; 4 SimTech, University of Stuttgart, 70565 Stuttgart, Germany

**Keywords:** shape-memory polymers, physical aging, water-triggering, positron annihilation lifetime spectroscopy, viscoelasticity, modeling, thermo-rheological simplicity

## Abstract

Polymers that allow the adjustment of Shape-Memory properties by the variation of physical parameters during programming are advantageous compared with their counterparts requiring synthesis of new material. Here, we explored the influence of hydrolytic (physical) aging on the Shape-Memory properties of the polyetherurethane system Estane, programmed in repeated thermomechanical cycles under torsional load. We were able to demonstrate that physical aging occurred through water adsorption influencing the existing free volume of the samples as well as the functional properties of Estane. Dynamic Mechanical Thermal Analysis determined the glass transition temperatures of dry and hydrolytically aged samples. According to our results, Estane takes up to 3 wt % water for two weeks (at an ambient temperature of θ = 20 °C). The glass transition temperatures of dry samples decreased within this period from 55 to 48 °C as a consequence of a plasticization effect. Next, for both samples, six subsequent thermomechanical cycles under torsional loading conditions were performed. We were able to confirm that hydrolytically aged samples showed higher shape recovery ratios of *R*_r_ ≥ 97%, although dry samples revealed better shape fixity values of about 98%. Moreover, it was observed that the shape fixity ratio of both dry and hydrolytically (physically) aged samples remained almost unchanged even after six successive cycles. Besides this, the shape recovery ratio values of the aged samples were nearly unaltered, although the shape recovery values of the dry samples increased from *R*_r_ = 81% in the first cycle to 96% at the end of six repeated cycles. Further, the evolution of the free volume as a function of temperature was studied using Positron Annihilation Lifetime Spectroscopy. It was shown that the uptake of two other organic solvents (acetone and ethanol) resulted in much higher specific free volume inside the samples and, consequently, a softening effect was observed. We anticipate that the presented approach will assist in defining design criteria for self-sufficiently moving scaffolds within a knowledge-based development process.

## 1. Introduction

Shape-Memory Polymers (SMPs) encompass an exciting class of functional materials with the ability to be mechanically deformed and manipulated from a permanent shape to a temporary shape and retain this shape until an external stimulus is applied [1]. In the case of heat as the external stimulus, the polymer is referred to as a thermo-responsive SMP [1]. The structural elements of such polymers are permanent net points either of a chemical (e.g., in polymer networks) or physical nature (e.g., in thermoplastics) together with reversible crosslinks of switching domains that are related to a material-specific transformation temperature (often denoted as the switching temperature θ_sw_). In the case of amorphous SMPs, the glass transition temperature (θ_g_), and, in the case of semi-crystalline polymers, the melting temperature (θ_m_) are the respective switching temperatures. To assign the temporary shape, a programming process is conducted. This consists of heating the sample above its θ_sw_ and then mechanically deforming the sample to a temporary shape which is thereafter fixed by cooling to a temperature below θ_sw_. This leads to the fixation of polymer chains because of a sharp reduction in molecular mobility, and to adoption of the conformation set by the deformation. This temporary shape remains fixed even after the deformation stress is released. By heating the sample above the switching temperature θ_sw_, the Shape-Memory Effect (SME) is induced, and the permanent shape is triggered. The driving force for the SME is entropy elasticity of switching domains due to the stored elastic strain energy during deformation [2]. In the temporary shape, the switching segments are in an oriented conformation which then moves to a random coil-like structure by heating during recovery through entropy release. Investigation of the SME is typically performed in a thermomechanical cycle. Quantitatively, the kinetics and kinematics of Shape-Memory (SM) properties are measured in cyclic uniaxial tension or compression tests for small and large deformations, including further experiments like bending and twisting [3].

The resulting sets of torque-deflection and angle-temperature (M-φ-θ) data in torsion experiments can be used to calculate essential SM properties, e.g., shape fixation (*R*_f_), shape recovery (*R*_r_), maximum torque during recovery, and activation temperature of the shape recovery [4,5]. Shape fixity expresses the extent of retention between the applied temporary shape and the fixed shape after stress release at θ < θ_sw_. On the other hand, shape recovery is defined as the ability of the sample to recover its permanent shape [6].

It was observed that the functional properties of thermally-induced SMPs could be substantially influenced in the vicinity of solvents [7,8,9,10,11,12,13,14,15]. This finding motivated the concept of moisture-triggered SME [16,17,18]. Such polymers are of high technological relevance for biomedical applications such as surgical sutures or tissue engineering scaffolds temporarily substituting for the extracellular matrices [19,20,21]. The underlying mechanism is the lowering of the glass transition on account of the plasticization effect [10] through the dissolution of crystalline domains [22] or disintegration of hydrogen bonds [17] that happen to have been employed in triggering shape recovery in SMP systems. In all these cases, diffusion of solvents into the polymer matrices occurred after programming, leading to humidity-induced SMPs as reported by several researchers [20,21,22].

Besides two atomistic simulation studies reported recently [23,24], there is no systematic study concerning the effect of solvent uptake “before” programming on the functional properties of SMPs. Therefore, this contribution aims to survey whether solvent uptake leading to physical aging before programming influences the Shape-Memory capability of the commercially available polyetherurethane Estane (Lubrizol, Ovele Westerlo, Belgium). To that end, dry and physically aged samples were prepared. To physically age the probe examples, dry specimens were placed into three different solvents—Acetone (C_3_H_6_O), ethanol (C_2_H_6_O), and water (H_2_O)—For a defined period. Afterwards, their changes in specific free volume (FV) and related changes in θ_g_ were discussed by the mean of Positron Annihilation Lifetime Spectroscopy (PALS) and Dynamic Mechanical Thermal Analysis (DMTA).

The constructed dry and physically aged samples were subjected to a cyclic thermomechanical torsion test. This thermomechanical torsion test consists of twisting the samples up to φ = 360 degrees at a high temperature (θ_high_ = 70 °C), cooling down to a low temperature (θ_low_ = 10 °C) under controlled torque, unloading at θ_low_ = 10 °C, and stress-free recovery at θ_high_ = 70 °C. It was observed that physical aging through the incorporation of solvents, as mentioned earlier, into the polymer matrices remarkably changes the functional properties of Estane. Nevertheless, only the results for hydrolytically aged Estane will be presented here, as the amount of diffused acetone and ethanol into the polymer at the single steps of the Shape-Memory cycle could not be controlled. In this case, at the end of the experiments, unpredicted desorption effects were observed, and the data were not reproducible. However, for hydrolytically aged samples, no significant mass change was detected. It should be noted that the authors are currently trying to build a setup to perform SM cycles inside the respective media for two other aged polymers, so that desorption is prevented. Finally, the phenomenological modeling concept of Frozen Volume Fraction (FVF) was adopted to model the shape recovery behaviour of the dry and hydrolytically (physically) aged samples and to quantify the phase transition from the glassy state to the rubbery state concerning the influence of (i) water content and (ii) different heating regimes.

## 2. Materials and Methods 

### 2.1. Materials

For the experimental characterization performed here, a polyether-based thermoplastic polyurethane, commercially available under the name of Estane (Lubrizol, Ovele Westerlo, Belgium), was investigated without further purification. Estane is a block-copolymer, synthesized from methylendiphenylisocyanate (MDI) and 1,4-Butanediol with a polyether. For Estane, a number average molecular weight of about 132 kg/mol was reported using gel permeation chromatography (GPC) [25].

For solvent uptake and physical aging investigations, three different solvents were used: de-ionized water H_2_O (chemically pure, Kerndl, Weißenfeld, Germany), ethanol C_2_H_6_O (ACS-ISO, for analysis, purity: 99.8+%, Merck Chemicals, Darmstadt, Germany) and acetone C_3_H_6_O (ACS-ISO, for analysis, purity: 99.5+%, Merck Chemicals, Darmstadt, Germany).

### 2.2. Sample Preparation

For sample preparation, Estane granulates were processed using an injection molding machine (Arburg Allrounder 270M 500-210, Lossburg, Germany) with an injection temperature of about 204 °C and an outer temperature of the injection barrel of about 30 °C. Furthermore, the samples were molded with an injection rate of 26 mm/s, an injection pressure of 60 MPa, and a holding pressure of 55 MPa for 15 s. After processing, the molded plates were kept in a vacuum desiccator to keep them dry. Finally, prior to Shape-Memory experiments, DMTA tests, and PALS analysis, the samples were punched into rectangular samples with dimensions of W × H × L: 2 × 10 × 50 mm^3^ using a manual knuckle joint press. For the hydrolytic aging investigations, injection-molded plates of a thickness of 2 mm were cut to circular discs of diameter 44.5 mm with a mass of 9.777 ± 0.017 g.

### 2.3. Diffusion Experiments (Physical Aging)

For the diffusion experiments, dry Estane samples were settled in 200 mL de-ionized water, ethanol, and acetone baths under controlled temperatures. After specific periods of time, samples were taken out, dried by dabbing the wet surface with a wad of lint-free cotton, weighed, and then reimmersed in the reaction vessel. The weight gain during sorption experiments is described as the solvent mole uptake by 100 g of polymer:

(1) $$c_{t}:\;=c\left(t\right)=\frac{m_{s}}{\left(M_{s}\right)\times \left(m_{p}\right)},$$

where *m*_s_*(t)* is the evolution of mass of the sorbed solvent, *M*_s_ is its molar mass, and *m*_p_(*t =* 0) denotes the mass of the (dry) polymer sample in the initial state. The (time-dependent) relative weight gain can then be described as a function of time by

(2) $$a:\;=a\left(t\right)=\frac{c_{t}\;M_{s}}{1+c_{t}\;M_{s}}.$$

### 2.4. Dynamic Mechanical Thermal Analysis (DMTA)

Using rectangular samples with the mentioned dimensions of W × H × L: 2 × 10 × 50 mm^3^, Dynamic Mechanical Thermal Analysis (DMTA) experiments were accomplished in torsional mode with a double-motor rheometer equipped with an integrated Peltier-based environmental (temperature-/humidity-controlled) chamber (Anton Paar, Physica MCR 702 Twin Drive plus CTD 180, Graz, Austria). Here, the experiments were performed using a torque-controlled procedure. Moreover, the temperatures in the range of −5 to 120 °C could be adjusted and kept constant with a precision of ±1 °K.

A small uniaxial tensile force of around 0.5 N was superimposed to maintain the specimen under net tension. With a constant heating rate of 0.25 °K/min, temperature sweep tests with a prescribed amplitude (0.01%) and a constant frequency of 10 Hz were performed for both the dry and the hydrolytically (physically) aged samples. Such experiments provide valuable insights into the effective viscoelastic properties of the investigated polymer.

### 2.5. Cyclic Thermomechanical Twisting Tests

Thermoplastic elastomers like Estane have limited elastic deformation regimes. For such materials, torsional experiments are beneficial in comparison to uniaxial tests because they produce large isochoric shape changes, involving large nonlinear deformations (displacement/rotations), even at small or moderate strains [26].

For cyclic thermomechanical torsion tests, rectangular Estane samples with dimensions as mentioned earlier (cf. Section 2.4) were investigated. Shape-Memory cycles were conducted with the same shear rheometer described in Section 2.4. The humidity of the adopted environmental chamber for both the dry and the hydrolytically (physically) aged samples was controlled by a modular humidity generator (MHG 100, proUmid, Ulm, Germany), which can restrain relative humidity (r.H.) in the range of 10–95%. The described slender rectangular samples were fixed at the top and the bottom of the device through a prismatic joint. The specimens were twisted around their longitudinal axis by two motors positioned at the bottom and the top of the rheometer. Each cycle consisted of a Shape-Memory Creation Procedure (SMCP) under a rotational-angle-controlled mode and a recovery module. In the programming part, with a moderated heating rate of 3 °K/min, the samples were heated to the upper working temperature of θ_high_ = 70 °C and held at this temperature for 10 min to obtain a homogeneous temperature field. Following that, the samples were twisted to the maximum torsional deformation of φ_m_ = 360 degrees at a rate of 3.6 deg/s. Subsequently, under constant rotational angle conditions, the specimens were cooled down to a lower temperature of θ_low_ = 10 °C with a cooling rate of 5 °K/min and equilibrated for 10 min. Afterwards, the sample was mechanically unloaded at this temperature, and the temporary fixed torsional deformation φ_u_ was obtained along with the removal of stress. In the last step, recovery was induced by heating the specimen to the upper temperature under a stress-free condition with a heating rate of 5 °K/min. This cycle was repeated another five times to study the functional fatigue and effective material stability of the dry and the hydrolytically (physically) aged samples during successive SM cycles.

During cyclic torsion deformation, the quantities of the shape fixity *R*_f_ and shape recovery *R*_r_, expressing functional properties, can be calculated according to the following relations where *N* denotes the number of cycles [5,6]:

(3) $$R_{f}\left(N\right)=\frac{\varphi_{u}\left(N\right)}{\varphi_{m}}\times 100\%,$$

(4) $$R_{r}\left(N\right)=\frac{\varphi_{m}-\varphi_{p}\left(N\right)}{\varphi_{m}-\varphi_{p}\left(N-1\right)}\times 100\%.$$

Here, φ_m_, φ_u_, φ_p_ are the maximum deflection angle, the deflection angle after unloading, and the recovered angle, respectively.

### 2.6. Positron Annihilation Lifetime Spectroscopy (PALS)

Positron Annihilation Lifetime Spectroscopy (PALS) measurements were performed using a fast-fast coincidence spectrometer with a time resolution of 230 ps [27,28,29,30,31,32]. A positron source (22 µCi ^22^Na), wrapped in a 7 µm thick Kapton foil, was sandwiched between two identical samples of either dried or hydrolytically (physically) aged specimens. The samples were measured at room temperature or i–n the temperature range −50 to 100 °C. In each positron lifetime spectrum, 5 × 10^6^ counts were recorded. A silicon reference sample (218 ps) was measured for the source contribution, which was determined to be 14.7%. The software tool (LT 9) [33] was used to analyze the lifetime spectra, after the source and background corrections. 

PALS spectra are decomposed into three lifetimes, which are extracted using a nonlinear least-squares fit of a weighted sum of exponentials:

(5) $$N\left(t\right)=\overset{k+1}{\underset{i=1}{\sum }}\frac{I_{i}}{\tau_{i}}\exp\left(-\frac{t}{\tau_{i}}\right),$$

where τ_i_ denotes the lifetime of the positron state *i* and *I*_i_ is its relative intensity.

The first component (~0.15–0.17 ns) is due to the annihilation of Para-Positronium (P-Ps), the second (0.35–0.4 ns) is ascribed to the annihilation of free positrons, and the third (1.5–2.8 ns) is due to the pick-off annihilation of Ortho-Positronium (O-Ps). However, PALS is a well-established technique to be used for determining the free volume (FV) of holes (or cavities) in molecular materials [34,35], which is related to the lifetime of the O-Ps pick-off (τ_3_) by the Tao-Eldrup model [36]:

(6) $$\tau_{3}=\frac{1}{2}\mathrm{ns}{\left[1-\frac{R}{R+\Delta R}+\frac{1}{2\pi }\sin\left\{\frac{2\pi R}{R+\Delta R}\right\}\right]}^{-1}.$$

Here, *R* is the radius of the hole (potential well) and ∆*R* = 1.656 Å is the penetration depth of the O-Ps wave function into the material surrounding the potential well and represents the thickness of the electron layer. Specifically, *FV* = (4/3) π*R*^3^, in which *R* values are calculated from Equation (5).

### 2.7. Quantification of the Shape Recovery by Evolution Law for the Frozen Volume Fraction Model (FVF)

Commensurate with the phenomenological model of Frozen Volume Fraction (FVF), SMPs are composed of glassy and rubbery phases, whose FVF functions ϕ(θ) are internal state variables of the SM polymer and depend only on the temperature θ. Here, for the small strain investigations performed in our investigation, it was assumed that the total shear strain tensor (γ = 2ε_ij_, *i* ≠ *j*) with ε_ij_ = ε_ij_ $e_{i}⊗e_{j}$) is represented as the sum of two shear strain contributions from the frozen phase (γ_f_) and active phase (γ_a_) as follows:

(7) $$\gamma \left(t\right)=\phi \gamma_{f}\left(t\right)+\left(1-\phi \right)\gamma_{a}\left(t\right).$$

According to the free strain recovery experiments during heating, Liu et al. [37] suggested a phenomenological function which has the following form:

(8) $$\phi \left(\theta \right)=1-\frac{1}{1+c_{f}{\left(\theta_{\mathrm{high}}-\theta \right)}^{n}},$$

with two model-inherent material parameters *c*_f_ (1/K) and *n* (dimensionless) which must be determined from experimental observation. In addition, by considering the temporal development of frozen strain with temperature, Wang et al. [38] revealed that the FVF function has a similar form to the crystallization process. Therefore, by modifying the Avrami equation [39], an alternative function has been suggested:

(9) $$\phi \left(\theta \right)=\alpha \left(\exp\left[{\left(-\frac{\theta_{t}}{\theta }\right)}^{m}\beta^{n}\right]\right),$$

where α is the FVF, β is a dimensionless cooling rate, and *n* is the Avrami exponent. 

The next model was introduced by Kazakeviciute-Makovska et al. [40]. They have analyzed different FVF functions (ϕ(ξ)) and suggested a modified general function based on a reduced, i.e., dimensionless temperature ξ, depicted by the following equation:

(10) $$\phi \left(\xi \right)=\frac{1}{1+\exp\left[c_{0}\left(\xi -\left(1-b_{0}\right)\right)\right]}.$$

In contrast to Equations (8) and (9), the model of Kazakeviciute-Makovska et al. [40] is proposed in a completely dimensionless setting. Again, *b*_0_ and *c*_0_ are two material-specific (dimensionless) parameters, and ξ(t) is the dimensionless reduced temperature in terms of the transformation temperature θ_t_ obtainable from experimental investigations:

(11) $$\xi \left(t\right)=\frac{\theta \left(t\right)}{\theta_{t}},\;\xi_{\mathrm{high}}=\frac{\theta_{\mathrm{high}}}{\theta_{t}},\;\xi_{\mathrm{low}}=\frac{\theta_{\mathrm{low}}}{\theta_{t}}$$

In spite of the model of Kazakeviciute-Makovska et al. [40], the physical meaning of the parameters involved in the phenomenological FVF models remain unclear. Further, in most cases, it is assumed that the strain recovery profile has a similar form to the proposed FVF function, although the shape recovery profile changes its shape for different types of SMPs, especially at moderate and large deformations. In the present contribution, in an attempt to predict the evolution of the deflection angles during recovery in cyclic thermomechanical torsion tests, a simple form of a double-logistic sigmoidal function was applied, in contrast to previous models. The proposed function is capable of elucidation of the influence of an extra programming parameter (e.g., amount of aging material or heating rates) on the functional properties of dry and physically aged samples. This specific FVF model, again proposed in a dimensionless setting, is a generalized form of the previously mentioned functions and can be described as

(12) $$\phi \left(\xi \right)=1-\left(\frac{\varphi_{p}}{\varphi_{u}}-1\right)\left[\frac{a}{1+\exp\left(c_{1}\left(\xi -b_{1}\right)\right)}\right]+\left[\frac{1-a}{1+\exp\left(c_{2}\left(\xi -b_{2}\right)\right)}\right].$$

Within this dimensionless form of the evolution law for Frozen Volume Fraction, it is now possible to study the basic characteristics of dry and hydrolytically (physically) aged samples in a comparable manner. In Equation (12), the proposed constitutive form is adopted to torsion experiments of beamlike samples with the angle ϕ as the primary kinematical quantity. The parameter *a* in Equation (12) is a dimensionless mass percentage of existing water inside the sample, as defined previously (cf. Equation (2)). The quantity φ_u_ is the unloading angle at the end of unloading stage, and φ_p_ is the recovery angle at the end of recovery (cf. Equations (3) and (4)). Moreover, *c*_1_ and *c*_2_ are both dimensionless material parameters determining the speed of transition from glassy to an amorphous state, and *b*_1_ and *b*_2_ identify the shift factor of recovery to higher or lower temperature values.

## 3. Results

### 3.1. Solvent Uptake Experiments

**Figure 1:**
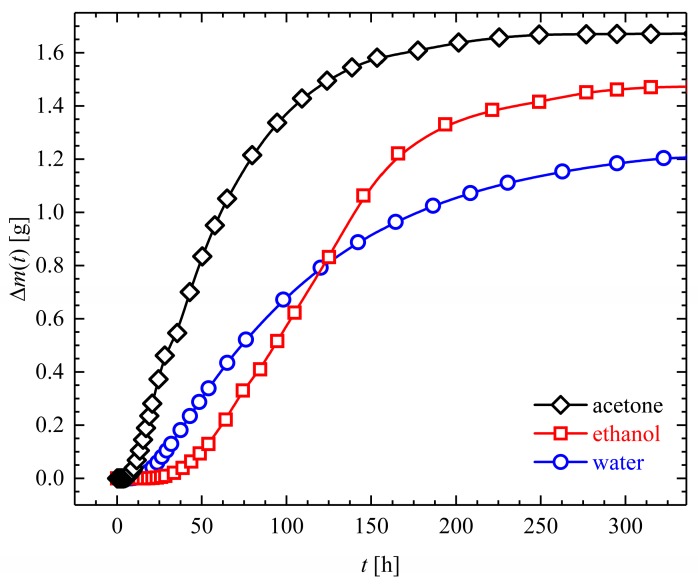
Temporal changes of the mass of Estane sample (with dimensions of W × H × L: 2 × 10 × 50 mm^3^) exposed in water, ethanol, and acetone baths at an ambient temperature of θ = 20 °C. Comprehensive weight gain results of Estane immersed in different solvents (water, ethanol, and acetone) at different temperatures can be found in [25].

The temporal mass gain of Estane samples immersed in three different solvents (water, ethanol, and acetone) at θ = 20 °C is depicted in Figure 1. The maximum amount of solvent which can be uptaken by Estane is called the saturation value for increase of mass and is denoted by $m_{\infty }$. The solubility of water in Estane is very limited and after a period of two weeks, Estane is fully saturated with water. A saturation value for increase of mass developed over the course of the experiment was measured to be around 3 wt % for water. However, clearly much higher amounts of ethanol and acetone—Up to 15 and 35 wt %, respectively—Can be taken up by the specimen.

As can be observed from Figure 1, the absorption kinetics of individual solvents into Estane at θ = 20 °C are different. Here, several parameters, e.g., molecular mobility of the solvents inside polymer matrices, their solubilities, and or elastic modulus of the entangled networks, play a significant role. It should be noted again that, for thermomechanical experiments, only hydrolytically (physically) aged samples have been used. Probe examples aged with ethanol and acetone shown here and in Section 3.3 are solely for comparison reasons and could not be used for SMCPs, as a significant amount of aging materials evaporates very quickly at every single step of the Shape-Memory cycle. 

In the next step, prior to thermomechanical torsion tests for dry and hydrolytically (physically) aged samples, two sets of experiments were performed: (i) temperature sweep tests of DMTA and (ii) PALS measurements. The sizes of the holes and their distribution inside the dry and physically aged samples as a function of temperature can be studied by PALS measurements. Moreover, with temperature sweep tests, interesting information about the structural properties of Estane could be attained.

### 3.2. Temperature Sweep Tests of DMTA

**Figure 2:**
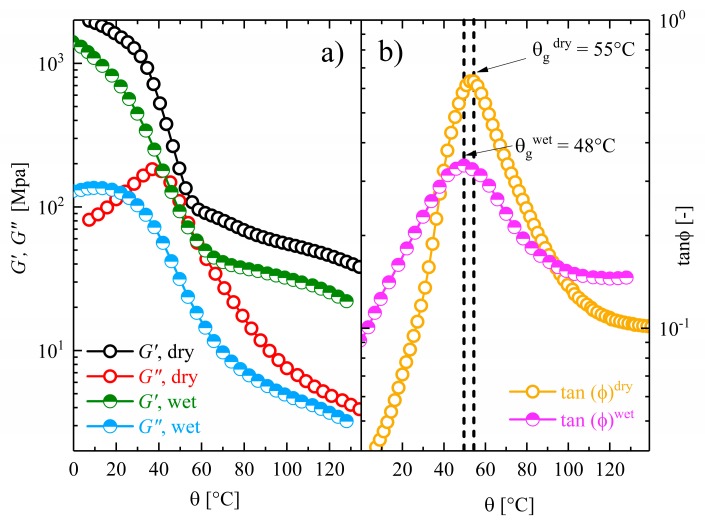
Comparison of the thermomechanical properties of dry and hydrolytically (physically) aged Estane obtained by DMTA experiments. (**a**) Change of storage and loss moduli of dry (empty circles) and hydrolytically (physically) aged samples (half-filled circles) with respect to the temperature and (**b**) changes of loss factor (tanϕ)^dry^ for dry (empty circles) and (tanϕ)^wet^ for hydrolytically (physically) aged specimen (half-filled circles) as functions of temperature.

The temperature dependencies of the storage modulus *G*′ and loss modulus *G*″ of Estane are depicted in Figure 2a. It can be observed that the storage moduli of dry and hydrolytically (physically) aged samples decrease gradually until the associated glass transition temperature $\theta_{g}$ is reached. The loss factor (tanϕ)^dry^ of dry samples increases progressively up to its maximum, where the rheological material’s dissipation energy is at supremum. This behavior is typical for physically crosslinked copolymers [6]. In contrast, the increase of the loss factor (tanϕ)^wet^ in the hydrolytically (physically) aged samples is less pronounced (see Figure 2b).

The glass transition temperature $\theta_{g}$ of the samples can be determined from the maximum of the tanϕ-θ-graph. It can be observed that, as expected, diffusion of water leads to a reduction in the glass transition temperature of up to 7 °C. This is relatively high for this small amount of water and may be attributed to the deterioration of physical bonds.

### 3.3. Study of Free Volume (FV) with PALS

**Figure 3:**
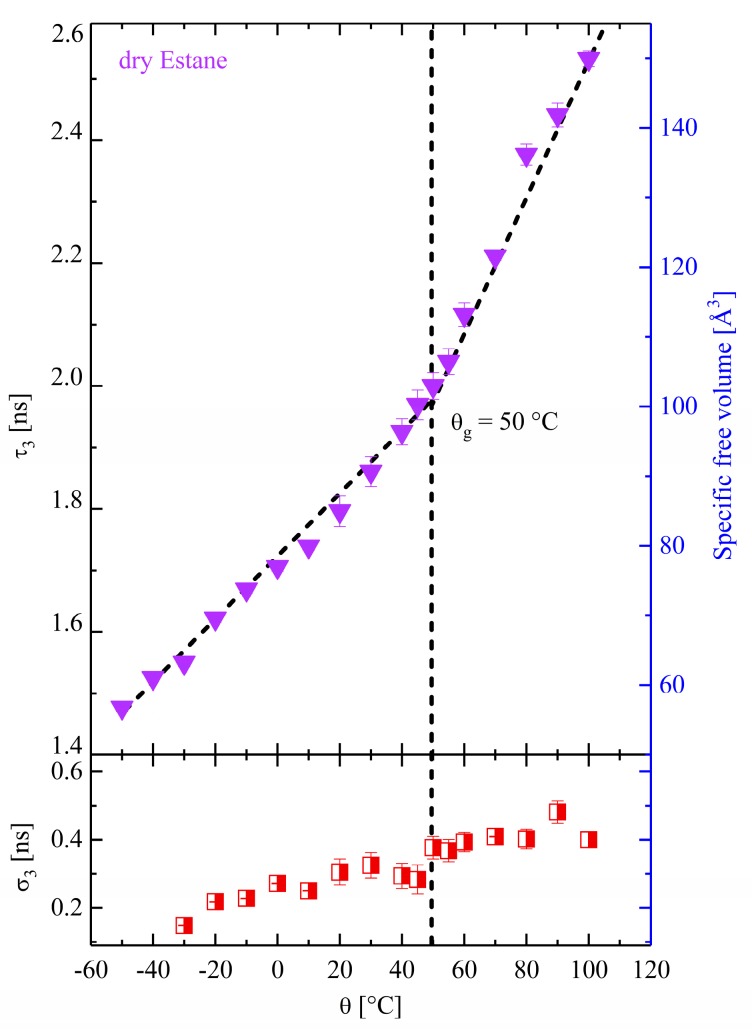
The temperature-dependent Ortho-Positronium (O-Ps) mean lifetime and its corresponding specific free volume in dry Estane (filled triangles). The standard deviation σ_3_ as a function of temperature is presented in the lower panel (half-filled quadrates).

The physically aged and dry specimens were measured in a vacuum in the temperature range of −50 to 100 °C using PALS. The lifetime spectra of all samples were analyzed in the dispersion mode and measured during increasing and decreasing temperatures. All parameters depicted here represent the average of two 4 h (each with 8 M total count) measurements. The data were well reproducible within the statistical errors of the experiments. The results of temperature-dependent PALS measurement ($\tau_{3},\;\sigma_{3}$, and FV) performed on dry Estane are displayed in Figure 3. The O-Ps pick-off annihilation lifetime and the calculated free volume are presented in the upper panel. The hole volumes are also plotted versus temperature (θ) in this graph.

The presented PALS data in Figure 3 show that $\tau_{3}$ (O-Ps lifetime) increases from 1.49 ns at θ = −50 °C to a value of about 2.55 ns at θ = 100 °C, indicating an increasing mean free volume. Here, a dispersion of 0.2–0.4 ns could be observed. The O-Ps lifetime ($\tau_{3}$) does not show any hysteresis. Furthermore, the calculated free volume diminishes with decreasing temperature from $\theta_{\mathrm{high}}$ = 100 °C to $\theta_{\mathrm{low}}$ = −50 °C. Below $\theta_{g}$, the decrease of the calculated free volume with decreasing temperature is less pronounced and produces a kink in the FV-θ curve. Here, nearly two linear ranges can be distinguished: the rubbery phase at $\theta >\theta_{g}$ with a large slope (representative of rubbery thermal expansion) and a glassy state at lower temperatures $\theta <\theta_{g}$ with a smaller slope. The specific hole volume in the dry specimen is in the range of [51, 150] Å. As clearly shown, the change in the O-Ps lifetime (and, thus, the free volume) is due to the rubber-to-glass transition. Here, a glass transition of about $\theta_{g}$ ≈ 50 °C could be determined, which is a little bit higher than the value obtained by DMTA (cf. Figure 2). However, since $\theta_{g}$ is extremely measurement-method-dependent, a quantitative one-to-one comparison is not possible.

**Figure 4:**
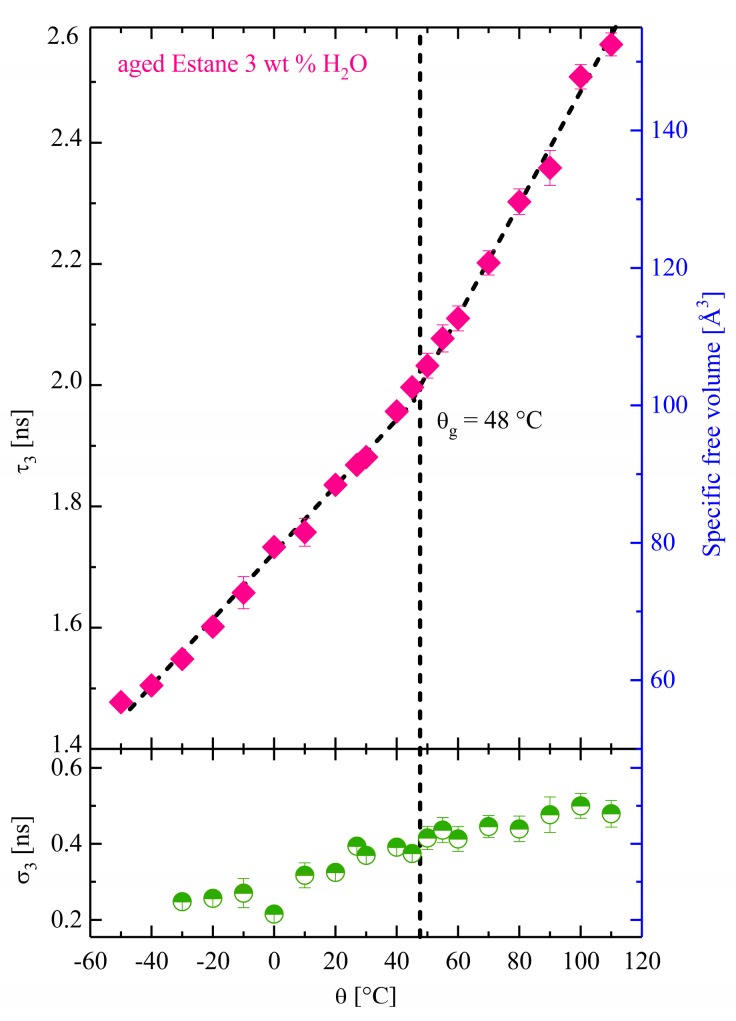
The temperature-dependent O-Ps mean lifetime and its corresponding specific free volume in hydrolytically (physically) aged Estane with 3 wt % H_2_O (filled diamonds). The standard deviation σ_3_ as a function of the temperature is presented in the lower panel (half-filled circles).

PALS results for the hydrolytically (physically) aged Estane with 3 wt % H_2_O are presented in Figure 4. Here, the samples show a glass transition temperature of θ_g_ ≈ 48 °C, and, thus, a glass transition lowering of about 2 °C. This agrees with the results of the DMTA tests and a hit for plasticization effects (cf. Figure 2).

According to the results shown in Figure 3, qualitatively similar behavior to that of the dry samples can be observed for hydrolytically (physically) aged Estane. As illustrated in this picture, in the temperature range between θ_low_ = −50 °C and θ_high_ = 100 °C, the specific hole volume in the hydrolytically (physically) aged samples varies between 53 and 154 Å. In the temperature ranges below $\theta <\theta_{g}$ = 48 °C, the hydrolytically (physically) aged samples are in the glassy state. The O-Ps detects pre-existing static holes. By increasing the temperature, the total free volume shows a weak thermal expansion which is slightly greater than that of the dry samples ($\alpha_{g}^{\mathrm{aged}}>\alpha_{g}^{\mathrm{dry}}$). In the rubbery phase, the molecular and segmental motion increase and the free volume holes obtain a more dynamic character and so the specific mean hole size distinctly increases as the temperature increases.

**Figure 5:**
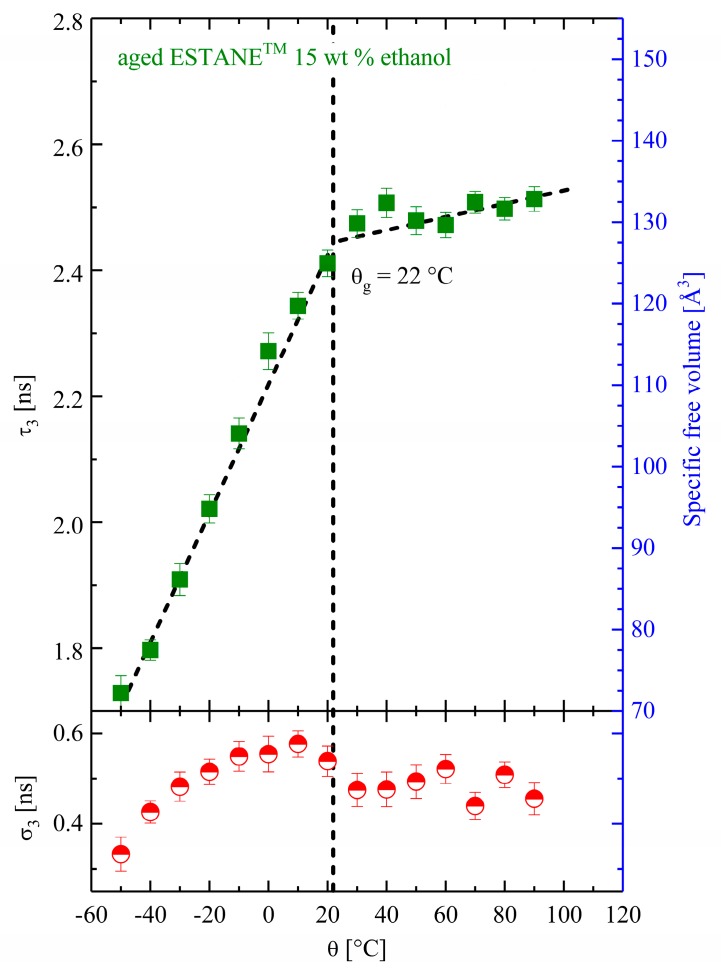
The temperature-dependent O-Ps mean lifetime and its corresponding specific free volume in physically aged Estane with 15 wt % ethanol (filled circles). The standard deviation σ_3_ is shown in the lower panel (half-filled circles).

**Figure 6:**
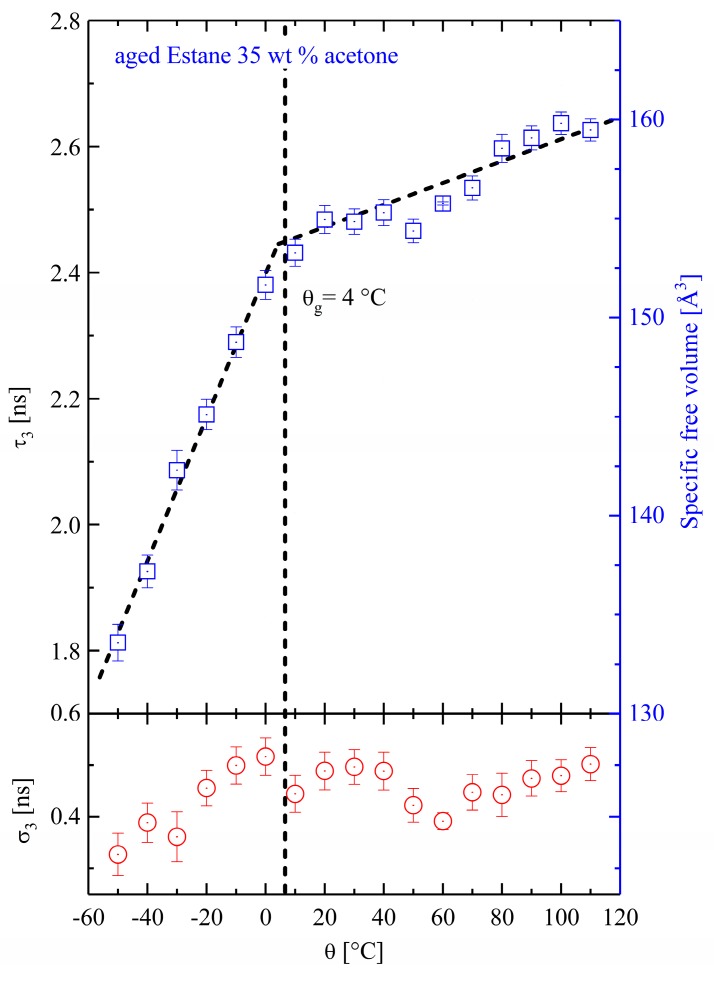
The temperature-dependent O-Ps mean lifetime and its corresponding specific free volume in physically aged Estane with 35 wt % acetone (empty quadrates). The standard deviation σ_3_ is presented in the lower panel (empty circles).

For comparison reasons, the temperature-dependent PALS measurements were performed for samples aged with two other solvents (Estane with 15 wt % ethanol and with 35 wt % acetone). The PALS results are shown in Figure 5 and Figure 6. These results are impressive from the point of view that the DMTA experiments could not be performed for samples aged with acetone and ethanol without evaporation.

As depicted in Figure 5, in contrast to those in the dry and hydrolytically (physically) aged samples, the increase in specific FV inside the ethanol-aged specimen is obviously faster so that the glassy thermal expansion below θ_g_ is much higher than in the other two cases. Moreover, above the estimated glass transition temperature, the rate of FV-increase declines visibly. Here, the hole volume varies from 73 Å at −50 °C to 150 Å at 90 °C, which is much higher than for the dry and hydrolytically (physically) aged samples. 

The changes in the O-Ps lifetime and specific free volume with respect to temperature are shown in Figure 6 for aged samples with 35 wt % acetone. Qualitatively, the changes in the O-Ps lifetime and FV with temperature for aged samples with acetone is similar to the behavior of the aged samples with ethanol. However, a much higher amount of specific FV can be observed quantitatively, indicating that the polymer chains are pulled apart from each other. Possible reasons are the higher amount of uptaken solvent and the larger size of a single acetone molecule (nearly 4.197 Å) compared to ethanol (about 4.134 Å) [41]. Here, the FV extends from 134 Å at −50 °C to 161 Å at 90 °C.

**Figure 7:**
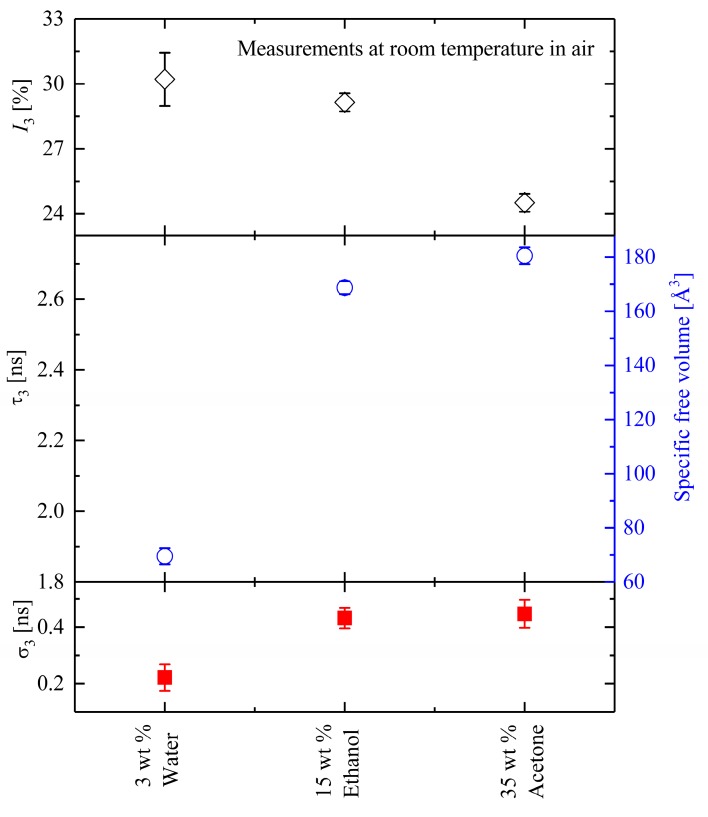
The O-Ps parameters measured in Estane under different treatments at θ = 20 °C. Upper panel: the variation of the intensity *I*_3_ during the measurements in hydrolytically (physically) aged Estane with 3 wt % H_2_O, 15 wt % ethanol, and 35 wt % acetone (empty diamonds). Middle panel: The O-Ps mean lifetime τ_3_ and its corresponding specific free volume (empty circles). Lower panel: the standard deviation (σ_3_) of the O-Ps in physically aged samples (filled quadrates).

The experimental results shown in Figure 7 were investigated in air at room temperature immediately after extracting the samples from the packing. The effect of the treatments is clearly shown. The O-Ps lifetime τ_3_—And, thus, the free volume (FV)—Increases with the treatments of the Shape-Memory samples and the intensity *I*_3_ declines, indicating that the population (concentration) of the holes decreases as the Estane is treated. This is a hint for the evaporation process. PALS results showed that the θ_g_ decreases with the amount and type of aging material. It could be demonstrated that the glass transition temperature of Estane decreased from 50 °C for the dry state to 4 °C for aged Estane with 35 wt % acetone. In all these cases, the existing specific FV inside the samples increased with the amount of aging material so that the samples with 35 wt % acetone showed a rubbery behavior at room temperature and change from transparent to opaque in appearance. Moreover, it should be mentioned that a direct correlation between the amount of uptaken aging materials and glass transition lowering was almost impossible since the uptake of acetone and ethanol inside Estane is too fast and 3 wt % uptake is exceeded very quickly.

### 3.4. Thermomechanical Cycles: Torsion Tests for Dry and Hydrolytically (Physically) Aged Samples

**Figure 8:**
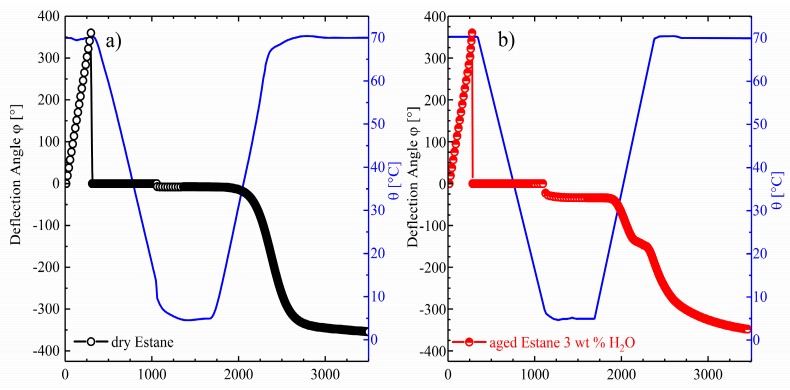
One complete thermomechanical test cycle (steps (i–iv)) under torsional loading as deflection angle φ(*t*) plotted for (**a**) dry (empty circles) and (**b**) hydrolytically (physically) aged Estane (half-filled circles). The corresponding θ-values are plotted as blue lines with respect to the right ordinate.

In this section, the functional properties of Estane under mechanical torsional loading are discussed. Figure 8 shows one complete thermomechanical cycle for dry and hydrolytically (physically) aged Estane. Results are presented as deflection angle (φ) versus time (*t*) plotted together with the respective temperature (θ), consisting of the four steps introduced in Section 2.4 of Materials and Methods: (i) heating and twisting by applying 360 degree torsion i.e., one full twist, (ii) cooling to θ_low_, (iii) unloading at θ_low_, and, finally, (iv) recovery during heating ramp to θ_high_. It should be noted that, in Figure 8, for both dry and hydrolytically (physically) aged samples after φ = 360 degree deformation, the deflection angle was set to zero degrees for a better observation of the results. 

**Figure 9:**
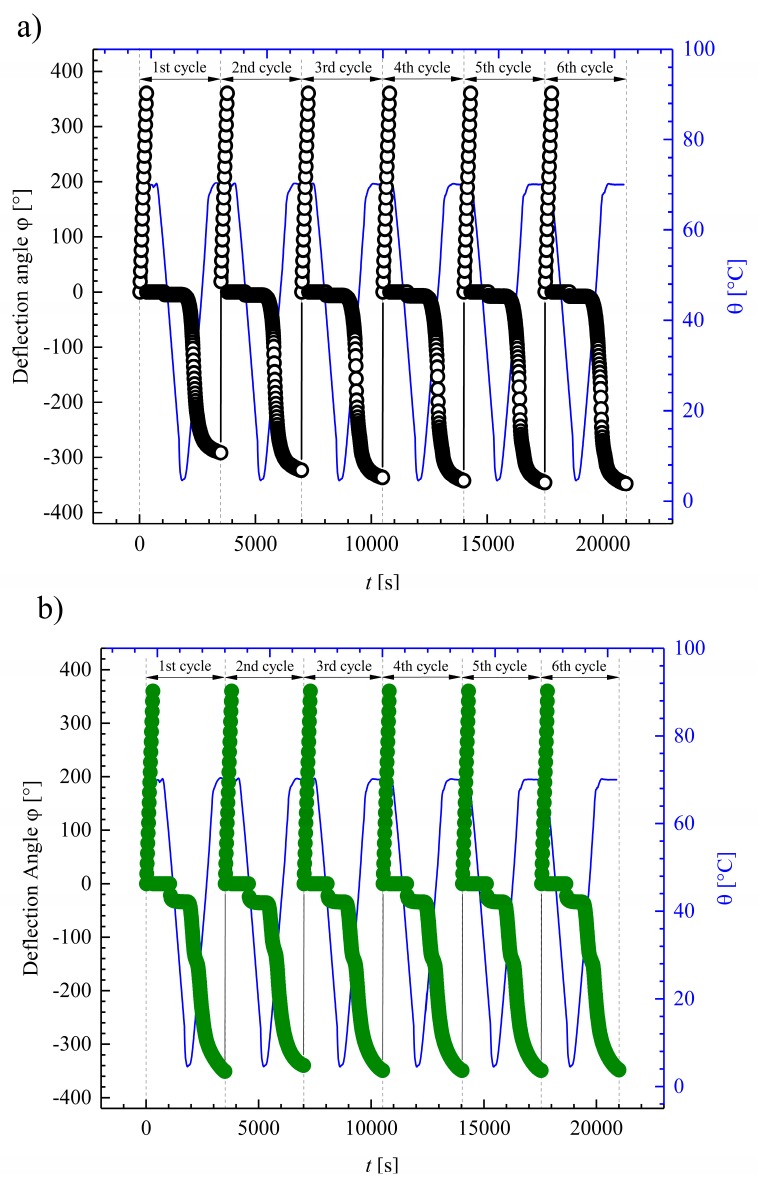
Six successive thermomechanical test cycles (steps (i–iv)) under torsional loading as deflection angle φ(*t*) plotted for (**a**) dry (empty circles) and (**b**) hydrolytically (physically) aged Estane (filled circles). The corresponding θ-values are plotted as blue lines with respect to the right ordinate.

In addition to Figure 8, six repeated thermomechanical cycles are illustrated for dry and hydrolytically (physically) aged samples in Figure 9. With the aid of these six thermomechanical cycles, it is now possible to study the influence of the repeating cycles and the functional fatigue on the Shape-Memory properties of Estane.

As depicted in the graphs of Figure 8 and in Figure 9, a qualitatively similar behavior can be observed for both dry and hydrolytically (physically) aged samples during programming, i.e., twisting, cooling, and unloading; however, a better apparent unloading capability can be noticed for the dry specimen.

**Figure 10:**
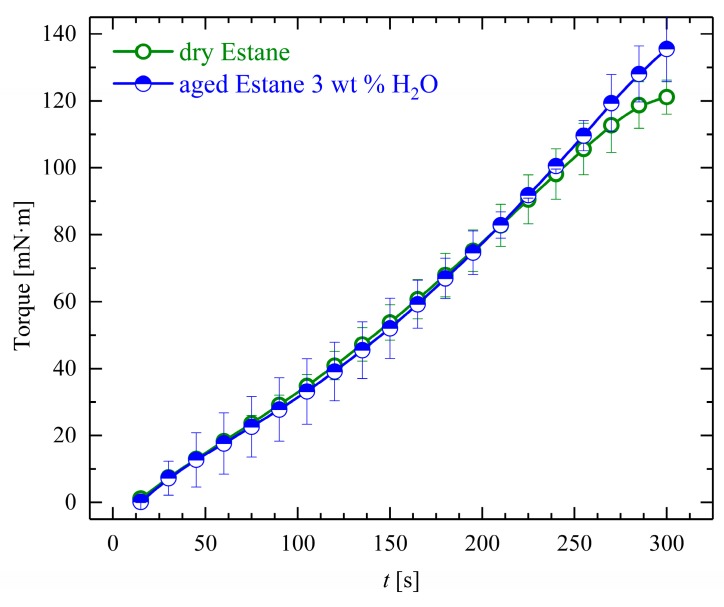
The evolution of the applied torque needed for one complete twisting φ = 360 degrees for both dry (empty circles) and hydrolytically (physically) aged Estane (half-filled circles).

During deformation at θ_high_, for both dry and hydrolytically (physically) aged samples, the obtained mean torque values overlap each other at the beginning of the deformation step, so that qualitative similar behavior could be observed. Interestingly, a slightly higher amount of torque was needed to twist the hydrolytically (physically) aged samples, as shown in Figure 10. 

Furthermore, the shape recovery of the samples in cycles N>1 is better than in the first cycle for both the dry and the hydrolytically (physically) aged specimens. However, the shape fixities remain almost unchanged. Moreover, as depicted in Figure 8 and Figure 9, the behavior of subsequent samples converges after the first cycle. Here, the first cycles behaved as a preprocessing procedure which led to the similar pre-orientation status of macromolecular chains in both the dry and the hydrolytically (physically) aged samples, independent from the amount of incorporated water. This means that the histories of the specimen and eigenstresses produced through the extrusion and injection molding have vanished after the initial cycle. 

**Figure 11:**
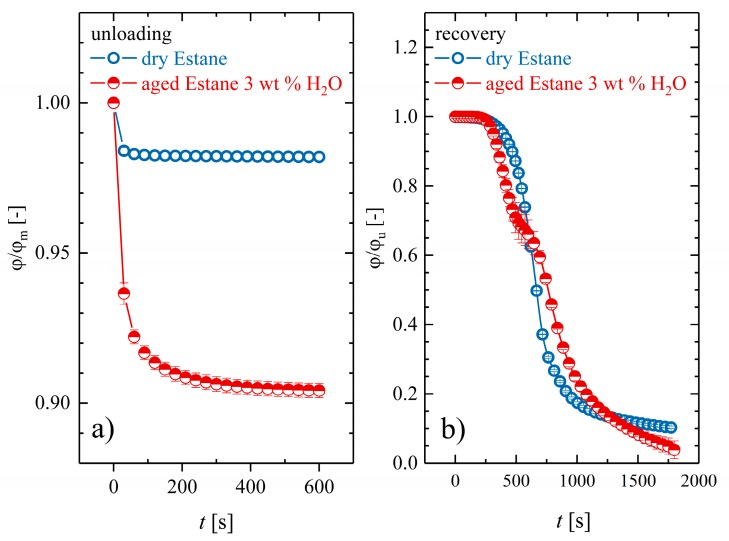
(**a**) The evaluation of the normalized averaged deflection angle values in relative units for the unloading step at θ_low_ = 10 °C for dry (empty circles) and hydrolytically (physically) aged samples (half-filled circles) and (**b**) the evaluation of the normalized averaged deflection angle values in relative units for the recovery step (right panel) at θ_high_ = 70 °C for dry (empty circles) and hydrolytically (physically) aged samples (half-filled circles).

In Figure 11a, the shape fixities of the dry and hydrolytically (physically) aged samples are depicted. Obviously, aged Estane shows a slower and inferior shape fixation capability because of the plasticization effect of low-molecular-weight compounds. Water molecules dissipate a part of the strain energy, which was stored during the preprogramming step as a result of the weakening of intramolecular forces through new hydrogen bonds. In Figure 11b, the recovery of dry and hydrolytically (physically) aged Estane is illustrated. It can be noticed that the recovery profiles of the dry and hydrolytically (physically) aged samples differ quantitatively from each other in shape and form. The recovery profile of dry samples has the sigmoidal form, which is characteristic for dual SMPs. The inflection point of this graph determines the time at which the transition temperature is exceeded. However, the recovery profile for hydrolytically (physically) aged samples has a double-logistic sigmoidal form, mostly characteristic of block copolymers with two different $\theta_{g}$, showing a triple SME [4,5,6]. Moreover, one can perceive that the recovery of the hydrolytically (physically) aged samples is faster than that of dry ones such that after 450 s, 35% of the permanent shape is recovered. Besides this, it is believed that the existence of water molecules causes such a double-logistic sigmoidal behavior. This means that either diffusion of water molecules outside the sample or displacements, delocalizations, and rearrangements of polymer segments and/or water molecules are the reasons for such a form of recovery. 

**Figure 12:**
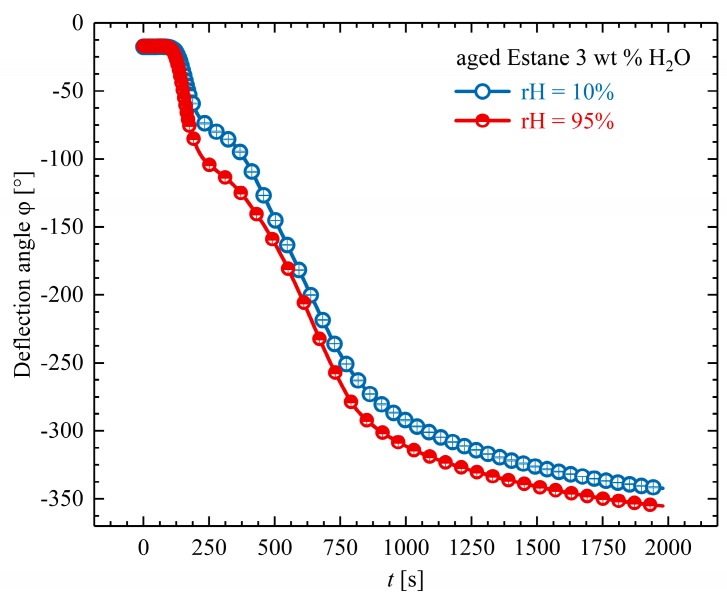
Temporal development of the averaged deflection angles φ for the recovery step at $\theta_{\mathrm{high}}$ = 80 °C under two different humidity conditions relative humidity (r.H.) = 10% (empty circles) and r.H. = 95% (half-filled circles).

To prove whether the diffusion of water molecules out of the specimen is the reason for this observation, we performed further SM cycles under different environmental conditions; in particular, the humidity in the environmental chamber (cf. Section 2.4) was adjusted and controlled to be around 95%. This was done to minimize the concentration gradients of the system and to reduce the probability of dehydration of the samples during programming and through recovery. It is worth noting that in all previous experiments, the humidity of the chamber was set to be 10% during the whole shape-creation and recovery process. Figure 12 shows the averaged temporal development of the deflection angles of the programmed hydrolytically (physically) aged Estane during recovery under the two different physiological conditions.

From Figure 12, it is evident that there is a minor difference between the recoveries of the hydrolytically (physically) aged samples under two different moisture states in humidity-controlled chambers. However, it is distinguishable that the recovery of the samples under 95% relative humidity condition is faster, and that they recover a higher amount of their original shape. According to these results, it can be supposed that water molecules do not evaporate from the samples even at arid conditions during this recovery time. This can be again explained through the creation of dominant H-bonds between water molecules and polymer chains. In hydrolytically (physically) aged samples, water molecules between hydrogen-bonded N–H or C=O form double hydrogen bonds with two already hydrogen-bonded C=O groups and also with other water molecules, leading to new 3D networks. Furthermore, the recovery of hydrolytically (physically) aged samples under higher moisture conditions may be a hint for surface adsorption between adjacent adsorption sites on the surface.

**Figure 13:**
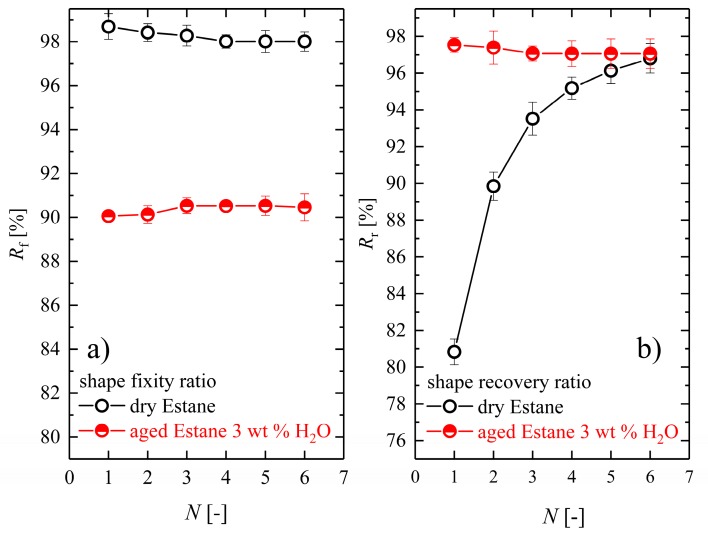
Comparison of the changes of (**a**) the shape fixity ratio *R*_f_ and (**b**) the shape recovery ratio *R*_r_ for dry (empty circles) and hydrolytically (physically) aged samples (half-filled circles) during six successive thermomechanical cycles.

Additional information about the quantity of SME in the successive cycles can be obtained from the shape fixity and shape recovery ratios calculated from Equations (3) and (4). During the unloading of dry and hydrolytically (physically) aged samples at low temperatures, the stresses were released, and the averaged instantaneous elastic angular recovery was measured. They were 5.04 and 34.20 degrees for the dry and hydrolytically (physically) aged samples, respectively. In Figure 13, the evolution of the shape fixity and shape recovery ratio for the dry and hydrolytically (physically) aged samples as a function of cycle numbers is depicted. It can be observed that the ability to fix the temporary shape during repeated torsional thermomechanical tests decreases slightly for dry samples, although shape fixity increases for hydrolytically (physically) aged samples. However, in the range of statistical errors one can generally interpret nearly constant shape fixity values for both classes as a constant fraction of the elastic recovery of about 1–2% for dry and 9–10% for hydrolytically (physically) aged samples. Additionally, it can be seen that the shape recovery *R*_r_ values of the hydrolytically (physically) aged sample do not change too much during the repeated cycles (97.6% in the first cycle and 97.2% in cycle number six), whereas for dry Estane *R*_r_, an increase from 81.1% to 96.7% was found. Likewise, at the end of the experiments, a shape recovery of about 324 degrees for dry and slightly better value of around 342 degrees for hydrolytically (physically) aged samples could be observed at the end of six cycles. We anticipate that these findings can be explained by the fact that the primary structural differences between un-deformed samples were no longer present after finishing the repeated cycles for both dry and hydrolytically (physically) aged samples.

Table 1 summarizes the results of shape fixation and shape recovery ratios at the end of the experiments *R*_r_(*t*→∞), *R*_f_(*t*→∞).

As listed in Table 1, within the statistical errors, one can perceive that the incorporation of water molecules into the dry samples caused a decrease in the value of the shape fixity, meaning that the amount of energy stored in the stretched hydrolytically (physically) aged samples is lower. Here, a shape fixity ratio of about 98% and 90% for dry and hydrolytically (physically) aged samples was calculated, respectively. Moreover, although for both dry and aged Estane a qualitatively similar progress during recovery was observed, the hydrolytically (physically) aged specimen exhibited not only a higher initial recovery rate but also a better shape recovery ratio of about 97% (compared to 90% for dry Estane). We attribute this result to the higher mobility of aged polymeric chains as a result of the increase in free volume.

### 3.5. Recovery Kinetics in Dry and Hydrolytically (Physically) Aged Samples

**Figure 14:**
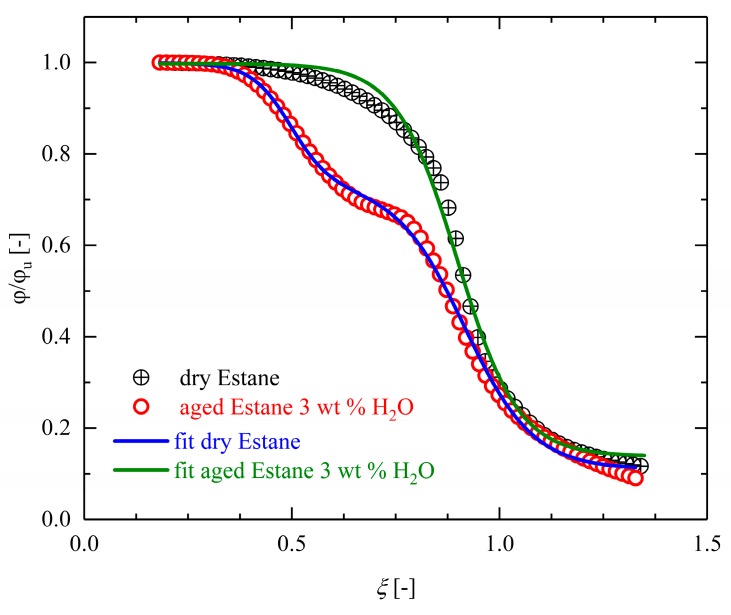
Development of the averaged recovery values of dry (open circles) and hydrolytically (physically) aged samples (half-filled circles) in relative units as a function of normalized temperature heated with a heating rate of $\dot{\theta }$ = $\frac{d\theta }{dt}=$ 5 °K/min and their corresponding data fit with the proposed Frozen Volume Fraction (FVF) model presented in Equation (12). The recovery profile of hydrolytically (physically) aged samples has a double-logistic sigmoidal form.

The experimental results of the evolution of the angle recovery of the dry and hydrolytically (physically) aged samples together with the results of the proposed FVF model (described in Section 2.7) are depicted in Figure 14. The fitted model parameters are listed in Table 2.

According to the results presented in Figure 14, our suggested FVF replicates the recovery behavior of both dry and hydrolytically (physically) aged samples very well. 

**Figure 15:**
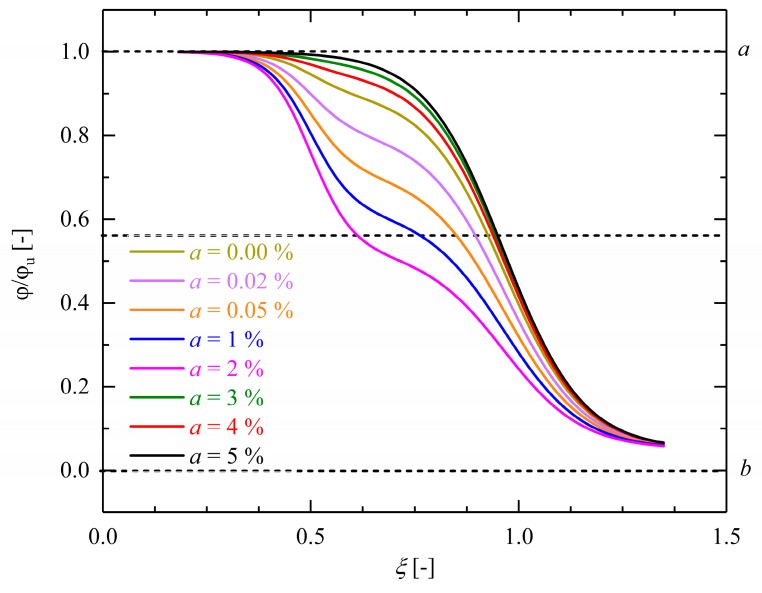
Influence of the material parameter *a* on the evolution of the angle as a function of the normalized temperature 𝜉 in relative units during recovery processes. The parameter *a* describes the mass percentage of existing water inside the samples (Equation (2)). An increase in *a* changes the appearance of the recovery profile from a single sigmoidal function to a double-logistic sigmoidal form. The black dash lines show the estimate transformation temperature and other asymptotic values of the suggested function in Equation (12).

**Figure 16:**
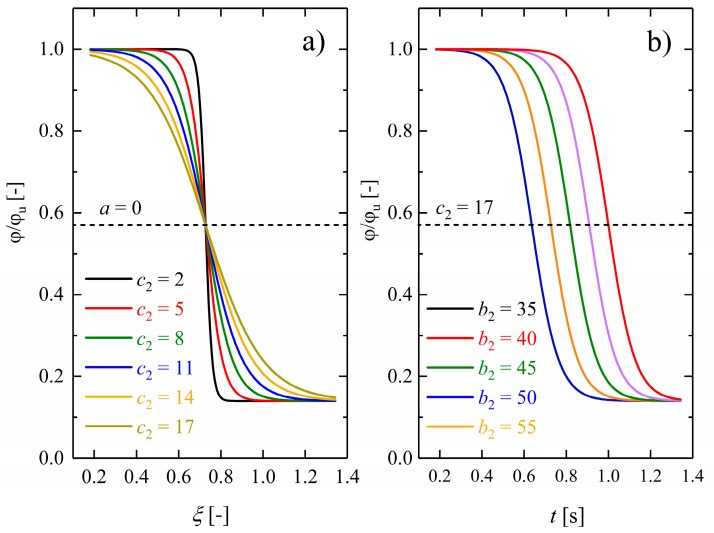
(**a**) The influence of the parameter *c*_2_, describing the transition rate from glassy to rubbery and (**b**) the influence of the parameter *b*_2_ (right), representing the shift of the recovery temperature because of different heating regimes on the shape recovery of dry Estane. The black dashed line shows the estimated transformation temperature. Other parameters are kept fixed.

As shown in Figure 15 and Figure 16, the predictive capability of this phenomenological model is improved by admitting involved material properties depending on experimentally observable/controllable parameters. Such properties are, e.g., the heating or cooling rate, or the mass percentage of the absorbed solvents influencing the shape and kinetics of recovery profile. The proposed dimensionless form of the FVF model makes it possible to study essential characteristics in a consistent way. 

According to Equation (12), the mass fractions of incorporated water molecules are described with the dimensionless parameter *a*. The influence of this dimensionless quantity on the shape recovery behavior of dry and hydrolytically (physically) aged samples is depicted in Figure 15. For dry samples, we set a=0. As in the case of hydrolytically (physically) aged Estane an amount of 3 wt % water was measured, the value a=0.03 was chosen. In Figure 15, evolving deformations are depicted as functions of temperature during the recovery step for dry (a=0) and hydrolytically (physically) aged samples up to 5 wt % water (a=0.05). It can be noticed that the mass fraction *a* is an important microstructural parameter which could be interpreted as an order parameter of the considered SMP. The observed double-logistic sigmoidal behavior of hydrolytically (physically) aged samples strongly depends on this quantity. Regarding Figure 15, it is evident that the qualitative and quantitative behavior of the function depend strongly on the values of the order parameter *a*. A higher value of *a* leads to larger initial recovery rates, meaning that the samples with more water content not only recover their original shape faster but also do so at lower temperatures. According to the results depicted in Figure 15, the relative evolution of the angles remains almost unchanged until the polymer matrices pass into the glass transition range. The inflection point of the graph can be therefore considered as the transformation (transition) temperature of the system. The transformation temperature of dry samples (*a* = 0) is around θ_t_ = 55 °C and is shifted to lower values because of the plasticization effect. As shown in Figure 15, a transformation temperature of θ_t_ = *b*_1_ = 27.41 °C (see Table 2) is noticed for samples with *a* = 0.03. However, a second, much higher θ_t_ can also be perceived (around θ_t_ = *b*_1_ = 50.47 °C). We attributed this behavior to delocalization and rearrangements of polymer segments and/or water molecules.

The influence of other material parameters on the form and speed of recovery is also illustrated in Figure 16 in relative units.

As exhibited in Figure 16a, the width of the functional form of the introduced double-logistic sigmoidal function is sensitive to the parameter *c*_2_. Polymers with a broad range of glass transition must have higher values of *c*_2_. From a physical point of view, the dimensionless quantity *c*_2_ explains the dynamics of chain segments close to the transition temperature. According to the results illustrated in Figure 16a, for low values of *c*_2_, the transition is much smoother than for higher values. In the case of semicrystalline polymers with the melting point as transition temperature, usually a relatively sharp transition is observed. In contrast, for amorphous polymers, the glass transition extends over a broad temperature interval. According to the results presented here, both of these cases can be very accurately described by the dimensionless form of the proposed Equation (12) with suitable values of the parameter *c*_2_. Finally, since the molecular motions engaged in the structural relaxation are roughly of the same sort as those involved in viscous flow, the relationship between *c*_2_ and the activation energy for structural relaxations can be correlated by the Moynihan relationship or determined experimentally by Differential Scanning Calorimetry (DSC) [42,43].

Moreover, the dimensionless parameter *b*_2_ modifies the previously introduced model [40], so that the predictive capability of the recovery profile is improved (cf. Figure 16b). The parameter *b*_2_ describes the width of the recovery profile, as exhibited in Figure 16b. As explained in Section 2.7, *b*_2_ is the second inflection point of the double-logistic sigmoidal function. From a physical point of view, *b*_2_ is itself a function of other experimental parameters like heating rates during recovery or similarly depends on the rate of change of any appropriate control parameter determining the transition of a stable or metastable equilibrium into the frozen-in, nonequilibrium state of the system [43]. Higher heating rates lead to higher recovery kinetics as a consequence of lower transition temperatures. Therefore, increased heating regimes resulted in a shift of recovery profiles towards lower temperatures.

## 4. Discussion

The measured glass transition temperatures for dry Estane (performed with DMTA experiments and with PALS measurements) agreed well with the results published in [44,45]. However, there are no experimental investigations about the influence of low-molecular-weight compounds on the glass transition of Estane. Although adsorption of small molecules (e.g., gases or water) does not depend upon FV, diffusion of such low-molecular-weight components is favored by local FVs. Recently, molecular dynamics simulations of a generic polymer model with finite extensible nonlinear elastic potential, which allows focus on generic rather than material-specific aspects, indicated that diffusion of small molecules leads to softening effects [46]. This is in striking agreement with the results published here. Other atomistic simulation studies on polyesters and polyester urethanes affirm this finding [23,24]. Further, the results announced in [46] showed that the particle size and shape of low-molecular-weight compounds have also a marked impact on the changes of glass transition temperature as the efficiency to use interstitial spaces decreases with increasing particle size. This is also interesting as acetone has the biggest and water has the smallest size [41].

The solvent uptake experiments performed here demonstrate that acetone shows the highest molecular mobility in Estane and water the lowest one. This agrees with the results of [25] and can be adequately explained with the generic model of [46].

A comparison of the torque needed for the deformation of dry and hydrolytically aged samples during successive thermomechanical cycles showed that the incorporation of water molecules into the polymer matrices leaded to an “effective” meantime stiffness. However, according to the performed DMTA results, this observation cannot be attributed to an antiplasticization effect. In contrast to our results, the antiplasticization effect of polyamide absorbed with water under medium and low relative humidity conditions was reported, which was a consequence of maximum loss of free volume of the sample up to half of the volume of a water molecule [47]. This finding was presumed to be an issue of firmly bonded water molecules and, accordingly, amide-amide bond displacements associated with highly strained chain conformation [47].

It was shown that the ability to fix the temporary shape during repeated torsional thermomechanical tests in the range of statistical errors remains almost constant for both dry and hydrolytically (physically) aged samples. Moreover, it could be demonstrated that the shape recovery values of dry Estane increases; however, no apparent change of *R*_r_ could be observed for the hydrolytically (physically) aged samples. These observations are very different from the literature and seem to be strongly chemical-structure-dependent. It was reported that, e.g., the shape recovery ratios of Tecoflex^®^ and Veriflex^®^, subjected to large uniaxial tension tests, decrease in cyclic thermomechanical cycles on account of an increase of irreversible strains [48,49,50]. Studies on poly(l-lactide) agree with this finding [51]. In contrast, the SME of a blend of polylactic acid and polybutylene succinate improves in repeated SM cycles [52] and remains nearly unchanged in polyimide systems [53].

## 5. Conclusions

In this work, the thermomechanical properties and functional properties of dry and hydrolytically (physically) aged Estane were studied by the means of DMTA and in successive thermomechanical cycles under torsion. The presented experimental results exhibited that the hydrolytic (physical) aging of Estane through inclusion of water molecules influenced its mechanical as well as its functional properties. The hydrolytic (physical) aging was found to result in different Shape-Memory behavior. Dry samples showed slightly better *R*_f_ values in comparison with those of the aged specimen, whereas higher *R*_r_ values, as well as faster recovery processes, were observed for the hydrolytically (physically) aged samples, which were not influenced by the number of cycles. Moreover, we demonstrated that the existing FVF models are a poor approximation in a series of systems in which small molecules are also influencing their functional properties. Here, we suggested the functional form of a double Boltzmann sigmoid curve, which was capable of addressing the influence of the number of water molecules on the shape and style of the recovery profiles of both the dry and the hydrolytically (physically) aged samples.

We demonstrated that the primary structural properties of Estane before Shape-Memory cycles are necessary to achieve a SME. However, these initial structural features can be influenced by physical aging. Among these structural properties are the specific existing holes or free volume elements distribution, which can be changed by the number of repeated cycles or the type and amount of aging material.

In accordance with the results exhibited here, we anticipate that these findings might motivate further studies on the impact of different aging materials and also the mechanism of the Shape-Memory behavior of polymers “prior to programming”, which could be extended to investigate the influence of resulting degradation products on the functional properties of SMPs. 

## Figures and Tables

**Table 1:** Shape fixity ratio *R*_f_ and shape recovery ratio *R*_r_ for dry and hydrolytically (physically) aged Estane during six successive thermomechanical cycles.

Sample	Shape fixity [%]	Shape recovery [%]
Dry Estane	98 ± 0	93 ± 6
Aged Estane 3 wt % H_2_O	90 ± 0	97 ± 1

**Table 2:** Material parameters obtained for the suggested Frozen Volume Fraction (FVF) model, cf. Equation (12), for dry and hydrolytically (physically) aged Estane samples.

Sample	φ_u_ [degree]	φ_p_ [degree]	*a* [-]	*c*_1_ [-]	*c*_2_ [-]	*b*_1_ [-]	*b*_2_ [-]
Dry Estane	5.1	324	0.0	-	14.28	-	0.89
Aged Estane 3 wt % H_2_O	34.2	342	0.03	0.37	0.23	27.41	50.47
